# Effect of a Multiaxial Load of Reverse Torsion on Open-Cell Aluminum Foams Behavior

**DOI:** 10.3390/ma16145136

**Published:** 2023-07-21

**Authors:** Solomon Bayu Huluka, Rachid Baleh, Naser A. Alsaleh, Adel Alfozan, Akrum Abdul-Latif, Sabbah Ataya

**Affiliations:** 1 Laboratoire Quartz, Supméca, 3, Rue Fernand Hainaut, CEDEX, 93407 Saint Ouen, France; solbayu2008@gmail.com (S.B.H.); rbaleh@gmail.com (R.B.); 2 Campus GARAC—Ecole Nationale des Professions de l’Automobile, 95100 Argenteuil, France; 3 Department of Mechanical Engineering, Imam Mohammad Ibn Saud Islamic University, Riyadh 11432, Saudi Arabia; naalsaleh@imamu.edu.sa (N.A.A.); afozan@imamu.edu.sa (A.A.); 4 IUT de Tremblay, Université Paris 8, 93290 Tremblay-en-France, France

**Keywords:** open-cell foams, multiaxial reverse torsion loading condition, mechanical behavior, energy absorption capacity

## Abstract

As a main goal of this work, a novel generation of cellular materials has been developed and manufactured by the kelvin cell model to be offered for different multifunctional applications. These Open-Cell Aluminum Foams (OCAF) have 85% porosities of spherical-shaped pores with a diameter of 11 mm. Several foamed square-section specimens were used. This work investigated the impact of different new quasi-static biaxial loading complexities on the mechanical behavior of such foams. Thus, new S-profiled rigs were already designed for examining the behavior of tested foams under biaxial loading conditions with different reverse torsional components named ACTP-S. After testing, their high specific strength and high energy absorption abilities have been characterized. Thus, in addition to the reference uniaxial test, all other tests were conducted at a speed of 5 mm/min. Thus, the mechanical responses of this foam are affected by loading complexities which are simple uniaxial, intermediate-biaxial (Bi-45°), and sever-biaxial (Bi-60°). These results were compared to the classical Absorption using Compression-Torsion Plastique (ACTP) responses. It was concluded that the highest dissipated energy increases with the increase in loading path complexity. Note that the energy absorption of the foam is essentially governed by its collapse mode.

## 1. Introduction

Over the past three decades, metal foams, as a new cellular material, have aroused considerable interest both from the scientific community and from manufacturing industries in all fields of engineering looking for breakthroughs and innovative solutions in terms of material selection.

Thus, they have been the subject of many investigations due to their microstructure and their mechanical behavior. However, multiaxial loading studies are relatively infrequent. However, understanding and controlling all the parameters governing their behavior remain essential for an optimized dimensioning of structures, particularly aluminum. Moreover, to be reliable, any design calculation should integrate, besides the type of foam, the strong variability of the behavior of foams in general. This is due to the coexistence of strong heterogeneities of the characteristics at various scales, macroscopic, cells, and finally microscopic (at the level of the cell walls and even below) levels.

In recent years, the study of metal foams has become an active area of research due to their desirable properties that can be used in diverse applications. This is undoubtedly due to their unique physical, chemical, mechanical, and thermal properties and wide promising applications (thermal insulation, heat sink for electronic devices, clean air technology, lightweight structural sandwich panels, energy absorption systems, etc.).

Commercially exploited and preferred for several applications due to their low density, excellent ductility, good thermal conductivity, and reasonable cost, they attract great interest [1,2,3,4,5,6,7].

Nowadays, foams based on alloys containing aluminum, copper, titanium, nickel, steel, and magnesium are widely used in the form of open cellular structures. They consist of solid metals and pores filled with a gas of high porosity [2]. Metal foams can be geometrically classified into closed-cell and open-cell foams depending on their topology, and their pore distribution can be classified as stochastic and regular. Closed cells are notable by the isolation of the neighboring cells from one another by a cell membrane; therefore, air and moisture are unable to get inside the foam. These are used principally as structural materials to support loads and absorb energy due to their excellent mechanical properties. Although, the cavities are interrelated in the open-cell foams as no face membranes separate the cells. These foams are used as (i) structural materials, (ii) functional materials in the fields of elevated-temperature filtration, (iii) sound and (iv) shock absorbers, and (v) heat dissipation due to their permeability or open porosity.

In most cases, aluminum-based foams are typically used in load-bearing elements where they may be subjected to multiaxial stress conditions [8,9,10]; to allow direct applications, a thorough understanding of their mechanical response under a multiaxial stress configuration (in particular combined shear-compression) is necessary. Studies have been performed in a modest number to characterize the effect of applied loading conditions on mechanisms of failure such as plastic collapse, buckling, and fracture.

Regarding aluminum foams, mechanical properties such as energy absorption capacity have been characterized under uniaxial [8,9,10] and multiaxial [11,12,13,14] loading conditions considering the strain rate, relative densities, and temperature. There are extensive studies under the uniaxial stress state, while limited documentation can be found under the multiaxial loading.

The behaviors of commercial aluminum foams have been examined by several researchers under multiaxial loading. Doyoyo and Wierzbicki [15] studied in detail the effect of pore size on the failure behavior of aluminum foam under biaxial loading. Zhou et al. [16,17,18] used the Arcan test rig. The latter was designed first to study the unidirectional responses of fiber-reinforced composites under multiaxial loading [19] which consisted of two pairs of semi-circular loading plates to study the fracture behavior of closed-cell aluminum foams under combined shear-compression loading case. Kossa [20] developed a special rig for testing polymer foam under biaxial compression. Two comb-like steel parts sliding into each other have been designed. A biaxial test rig setup to generate combined shear compression was used by Mosleh et al. [21], where two independent orthogonal compression and shear displacements are recorded simultaneously. Li et al. [22] employed two beveled-ended short cylindrical bars to produce the combined shear-compression loading [23]. Using these experimental procedures for multiaxial loading, significant experimental results of foams were collected. However, a somewhat simpler method is required.

It is worth noting that more information about the foam behavior under simple and complex loading conditions can be obtained by numerical simulations. Stress–strain responses of Duocel (open cell) and Alporas (closed cell) foams have been investigated by Deshpande and Fleck [24]. They studied the initial failure surface and its evolution using a compression load associated with a hydrostatic load. They showed that the hydrostatic yield strength controlled by the hydrostatic loading condition has a similar value to uniaxial yield strength. Furthermore, the stress–strain response of Alulight foams has been studied by Sridhar and Fleck [12] under uniaxial compressive loading simultaneously with hydrostatic pressure. It has been found that the hardening captured due to hydrostatic pressure exceeds that obtained under pure compression [25]. Moreover, the effect of triaxial loading on the fracture behavior of aluminum foams has also been examined [26]. It was assumed that the failure surface is a function of the third invariant of the stress tensor. For Duocel and Alporas aluminum foams, experimental failure responses were measured due to biaxial and triaxial loads [13]. Data with the three yield criteria for metallic foams have been compared. Miller’s criterion was retrieved from the yield surface for ideal open-cell foams considering the effect of cell wall curvature [27]. Deshpande–Fleck’s criterion was therefore developed except for a linear term in the mean stress [24]. The multiaxial failure of aluminum foams is suitably described via Miller and Deshpande–Fleck’s criteria.

**Figure 1:**
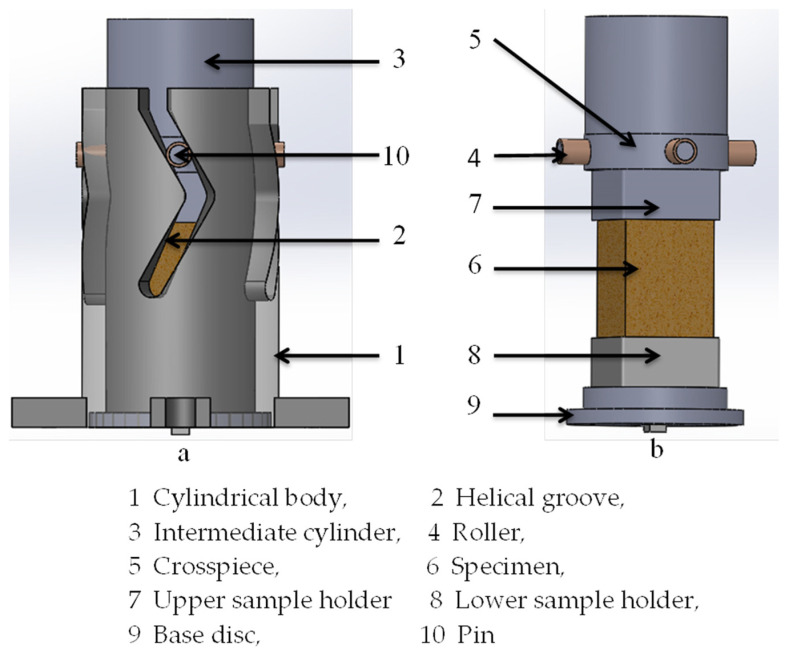
Three-dimensional presentation of the new variant of the biaxial device called ACTP-S showing (**a**) the assembly of foam and the fixation with the cylinder and (**b**) fixation of sample in sample holders before crushing.

The current work concerned the behavior of aluminum foams under biaxial loading paths. A combined compression-torsion loading over aluminum foam was generated using a specially designed rig shown in Figure 1 [28,29,30,31]. In this process, it is possible to produce several rates in the revered torsional component via a new extension of the ACTP rig. To study the effect of the reversal torsion component on the foam several propeller inclination angles (45°, 53°, and 60°) were designed, manufactured, and used giving rise to three new biaxial configurations named, respectively, S-Bi-45°, S-Bi-53°, and S-Bi-60°. For the tested metal foam, it can be perceived that increasing the loading complexity generated by ACTP will lead to an increase in the loading required to deform the foam. Thus, it could be easy to show that the most complicated loading condition is S-Bi-60°, giving us the highest foam strength compared to that of uniaxial loading [28].

To illustrate this extreme heterogeneity of the foam, different observation and analysis techniques are used (like the X-ray tomography technique) allowing the highlighting of the essential mechanisms characterizing the mechanical behavior of such foams where the localized plasticity and the failure of the cell walls occur.

## 2. Materials and Testing Procedure

The mechanical behavior of OCAF under such unprecedented loading complexity is studied and the result is compared to the classical ACTP rig results.

Open spherical cell aluminum foams having a porosity of 85% (respective relative densities of 15%), named FP-85, were tested. Their uniaxial response was considered as a reference.

With uniform pores with a regular shape and equal cell diameter, these foams were manufactured by regular sand casting as in [28], using the technique of replication involving 3 main steps: the open-pore pattern preparation via 3D sand printing, infiltration of the desired pattern and material, and lastly elimination of the pattern to recover the required foam. The Young’s modulus and tensile yield stress of the aluminum alloy are 74,000 MPa and 240 MPa, respectively. The chemical composition is summarized in Table 1. The morphology and pore distribution of the manufactured foam was investigated using X-ray tomography.

In order to approach this work, an aluminum foam was cut to the sample size (63 mm×63 mm×110 mm) by water jet. This metal-cutting technique is used to minimize the residual stress generated on the surface. To avoid foam deformation on the fixation, the foam specimens were reinforced by epoxy-risen at their two extremities. Then, the foams were loaded by a pure uniaxial compression and biaxial regime using the new variant (ACTP-S) of ACTP rigs developed in our laboratory. The yield stress, stress plateau as well as dissipated energy during the compression, and compression-torsion deformation of the various foam samples were determined and then compared.

A universal tension-compression machine (Instron 5582) is used, supplemented by the rig for the 3 biaxial configurations (45°, 53°, and 60°). This machine has a loading capacity of 100 kN having a range of cross-head speeds of 0.001–500 mm/min with the option of a speed jump. Identical experimental conditions are systematically used, under the same cross-head speed of 5 mm/min. The crushing of the structures is carried out as usual on this machine, between its two platens by a displacement of the top towards the bottom of the higher mobile platen. The protocol of this test campaign is summarized in three and/or five repetitive tests, which are carried out systematically for each configuration. The other test is necessary for any dispersion exceeding 5% of the results of the first two. It should be noted that thanks to the reliability of the experimental device and the scrupulous respect of the instructions relating to the experimental conditions inherent in each of the configurations, the two verification tests were carried out relatively little.

### 2.1. Uniaxial Tests

**Figure 2:**
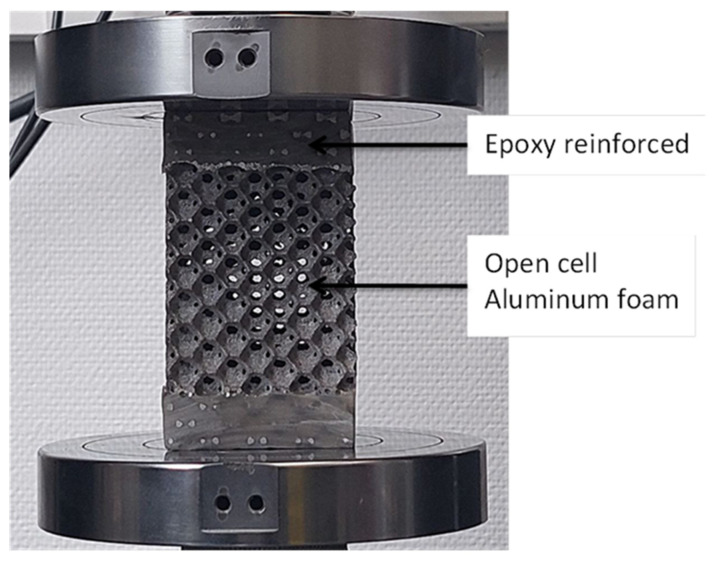
Uniaxial reference test on a specimen with extremities protected by an epoxy resin coating and remaining insensitive to any plastic deformation or buckling.

Under compressive load, square foam specimens were tested using an Instron 5582 Universal Materials Testing Machine. The specimen has effective dimensions of 63 mm × 63 mm × 70 mm. These specimens were loaded between two circular steel compression platens (Figure 2). All the tests were made using a speed of 5 mm/min. The sensed signals (displacement and load) were collected by the Instron machine software, Bluehill^®^ 2, version 2.35 throughout the crushing process.

### 2.2. Combined Compression-Torsion Tests

**Figure 3:**
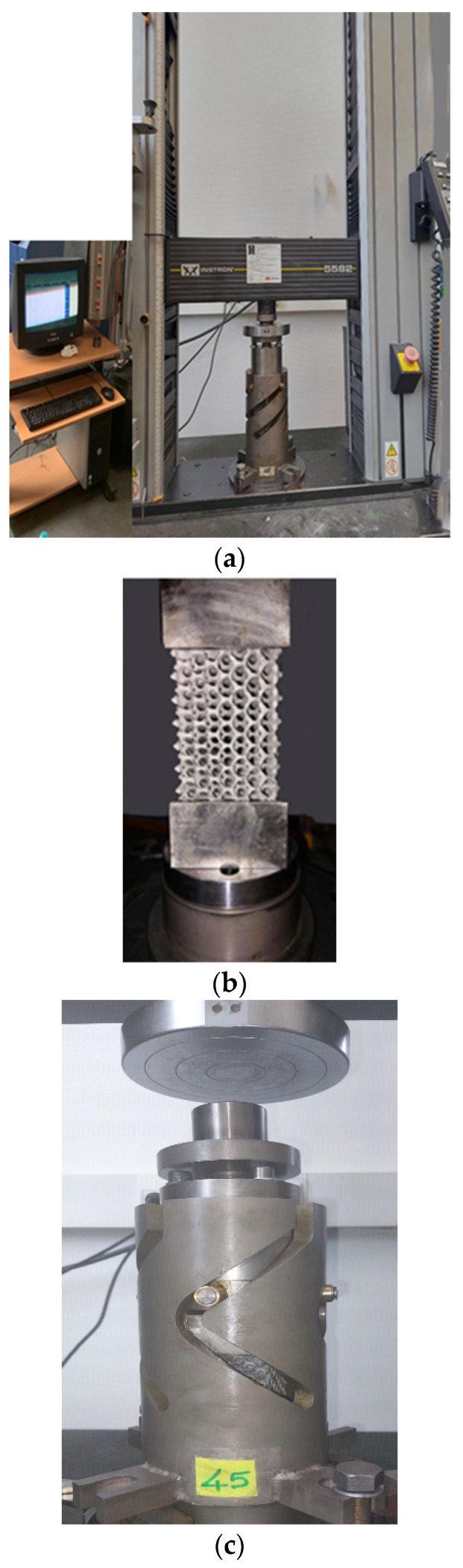
INSTRON Machine employed for biaxial testing and an illustration of the execution of the two biaxial tests: (**a**) classical via ACTP, (**b**) sample assembly, and (**c**) extreme biaxial via ACTP-S.

The aluminum foams’ deformation mode and yield behavior were studied using a combined compression-reverse torsion load. These experiments were conducted by employing the S-profiled ACTP rig, as shown in Figure 1 and Figure 3. The fundamental role of this rig is to provide the possibility to test material and structure by compression-torsion, such as crushing of metal foams, as already demonstrated in [28,29,30,31,32]. The goal of this rig is to transform the external unidirectional load into multidirectional load components [28,29,30,31]. The new rig used in this study generates a reverse compression-torsion loading configuration. The S-profiled rig can be described by the concurrent combination of axial compression and torsion. This S-profiled rig (Figure 1) consists essentially of a hollow tempered steel cylindrical body (1), in which four parallel S-shaped helicoid machined grooves (2) were machined and characterized by an inclination angle controlling the rate of change of the torsional component during compression. The effect of this parameter on the behavior of the specimen was studied using three different interchangeable cylindrical bodies of 45°, 53°, and 60°, respectively. The helicoid grooves receive a crosspiece (5) having four pivots (10) equipped with rollers made from bronze (4) guiding it in its movement of descent by instilling a rotational movement. This mechanism allowed the transformation of an initial uniaxial external load into a biaxial combined compression-torsion one. The bronze rollers have a role in minimizing the friction between the grooves (2) and the crosspiece (5). The deformed foam has a square prism form (6). On the bottom part of the sample holders, threaded holes were provided to fasten the bottom sample holder (8) to the base (9) and to fix the top sample holder (7) with the top guide (3).

The frictional effect on the crushing process was minimized by using after some tests new brass rollers as well as grease after each test. The evaluation of its effect on the absorbed energy was already evaluated in previous works [29,30,31]. Its variation, which depends on the angle of inclination (i.e., loading complexity), has an average of roughly 8%.

## 3. Results: Mechanical Properties

### 3.1. Uniaxial Plastic Flow

**Figure 4:**
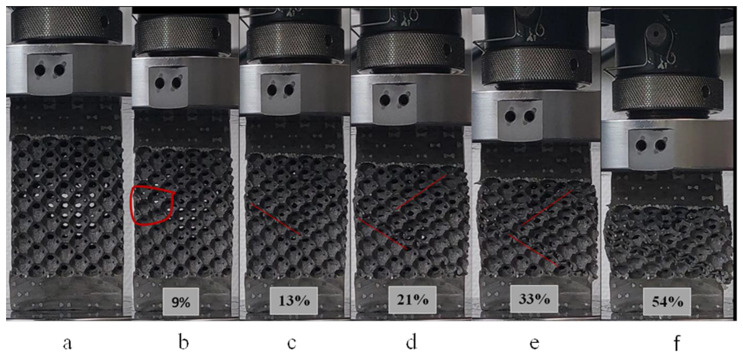
Successive closure of pores starting at the weak zone and progressing throughout the gage length under quasi-static uniaxial loading, (**a**) 0%, (**b**) 9%, (**c**) 13%, (**d**) 21%, (**e**) 33%, and (**f**) 54% defoemation, (percentages being axial strains).

The continuous observation of the crushing test through the taking of photographic images progressively to the crushed sample (Figure 4) indicates unambiguously, and following the literature, that this foam is incapable of deforming homogeneously. On the other hand, the random localization and centering of the plastic deformation as well as the propagation of the deformation bands, in parallel with the onset of the failure phenomena, are revealed as the main mechanisms characterizing the local mechanical plastic buckling behavior of this foam.

### 3.2. Cell Morphology and Pore Size Distribution

X-ray tomography has been used to investigate the 3D structure of the specimen before mechanical testing in a non-invasive and non-destructive way. It helps for a clear understanding of the internal local structure without destroying the sample.

**Figure 5:**
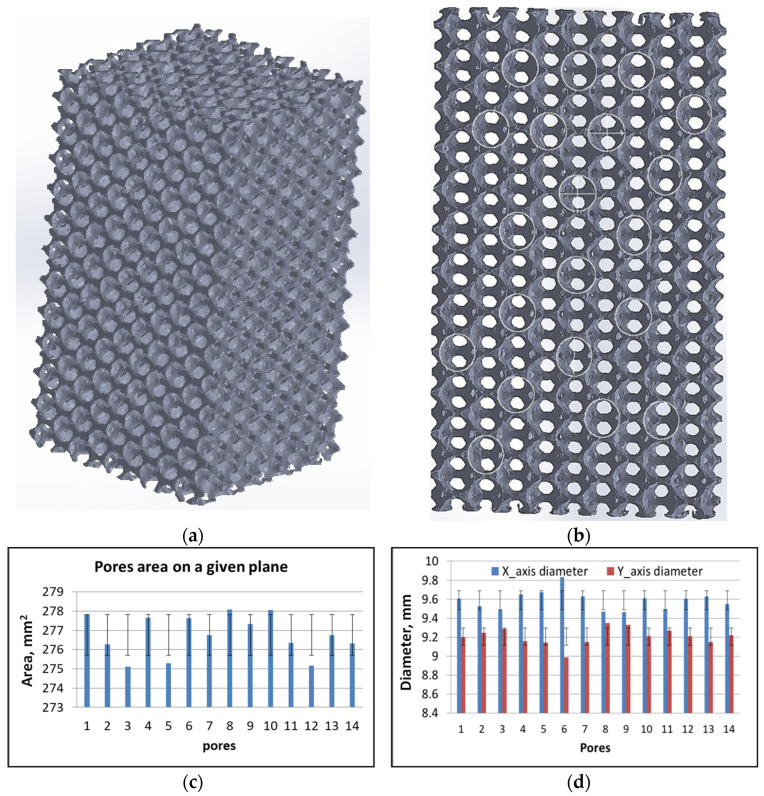
X-ray tomography image: (**a**) 3D image of the unreformed sample, (**b**) planner image to calculate the area of the pore using ImageJ 1.8.0, (**c**) bar charts representing the area of the pores, and (**d**) bar charts representing the major and minor diameter of pores.

Figure 5a,b shows the X-ray tomography of the undeformed sample as received. It was observed that the pores are equal in size and uniformly distributed. The distributions of the struts, the pores, and other features have been achieved through X-ray tomography which provides the reliability of the manufacturing techniques. The areas, major and minor diameters of the pores along specific planes were calculated using ImageJ 1.8.0, software (Figure 5c,d). The area standard deviation of 1.0 and diameter standard deviation of 0.1 along the x-axis and 0.0 along the y-axis were obtained. Such values elucidate that the pores have an almost similar diameter on the given plane.

### 3.3. Uniaxial Compression

**Figure 6:**
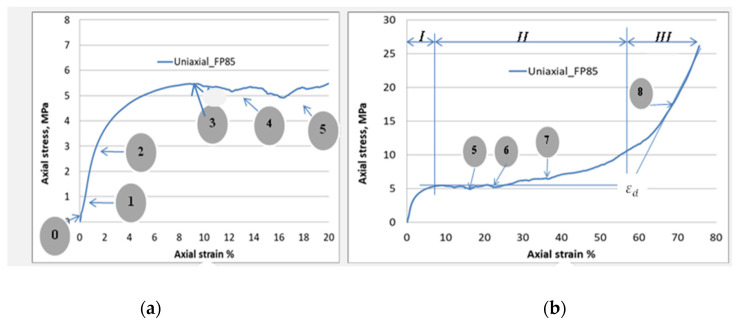
Engineering stress–strain curve under uniaxial compression loading. (**a**) initial region of the stress–strain curve up to strain = 20%, and (**b**) stress–strain curve up to fracture.

Figure 6 exhibit a stress–strain curve describing the mechanical response of the aluminum foam under uniaxial compressive load. It is worth noting, as adopted by other published works, e.g., [28,32], that the stress–strain curves were defined using the nominal cross-section of the specimen for stress and the nominal effective length for strain regardless of the percentage of porosity of the foam used. Different stages regarding this curve have been depicted in Figure 6b, where the distribution of strains is observed in the direction of the applied load. Concerning the stress–strain relationship, it can be characterized by three distinct zones: (I) pre-plateau zone (or elastic region), (II) plateau zone, and (III) post-plateau zone (after the densification process). Once the loading begins, a linear increase in stress as a function of strain is observed leading to an almost uniform strain. Moreover, this homogeneous deformation occurs for a limited value of compressive strain—up to 1.5% (Figure 6a). Then, there is the first localized strain (point 2) during which the slope of the stress–strain relationship decreases to the local maximum stress (peak stress) (point 3). Successive photos have been taken at different axial strains, as demonstrated in Figure 4. Indeed, strain bands were first generated by a 9% strain in the zone indicated by the red loop (Figure 4b). It is known that the crushing of foams occurs through the generation of successive crushing bands on the weaker zone. The photos taken (Figure 4) at each strain were referred to with the stress–strain curve (Figure 4). On this curve, for this example, the first band collapse appeared at such a strain (i.e., 9%); then, the red loop was placed at the given position to show the band failure. This can be interpreted by the presence of a locally weaker foam structure. With continued compressive stress, strains within the band and adjacent cell layers progress to local strains of 15–40%. This mechanism would be due to the widening of the strain band. Additionally, the rest of the specimen also participates in deformation. We can observe, in Figure 4a, with a strain amount of 33%, that the localization of plastic deformation (i.e., plastic bands) becomes less.

At the end of zone I, when a stress level is found at point (3) (Figure 6), zone II begins. This is characterized by an increase in the long relative stress plateau with an almost small slope due to a low-strain hardening (Figure 6b). Additionally, the phenomenon of serrations is present due to the local collapse of struts and cells. It is obvious that if more simultaneous cells have collapsed, a greater stress drop can consequently occur, as confirmed by points 5 and 6 of (Figure 6). For a strain range of nearly 15%, it is evident that several cells collapse at the same time, thus generating a second band of strain. When the crushing of the foam is carried out between 21% and 33%, practically all the deformations occur, and the bands of plastic strain invade the adjacent cell layers (Figure 4). At and after a compressive strain of 21%, the evolution of certain bands stops, while others begin to be generated. The zones already generated during zone I could be favored to create new deformation bands (Figure 4).

### 3.4. Biaxial Loading Configurations

**Figure 7:**
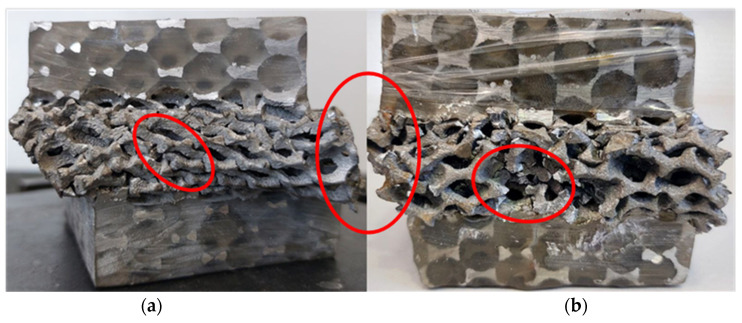
Comparison of deformed samples under two biaxial cases (**a**) classical Bi_45° and several (**b**) S-Bi-45°.

Figure 7 shows two typical examples of the evolution of plastic flow for the two classical and alternating biaxial configurations Bi-53° and S-Bi-53°. The red ellipses show the difference in plastic collapse band between the samples deformed using the classical ACTP rig (Figure 7a) and the new ACTP-S rig (Figure 7b). Indeed, observation reveals a more regular and pronounced deformation band with higher densification of the foam for the Bi-53° case. As for its counterpart S-Bi-53°, the localized deformation is less regular with the persistence of pores barely solicited. Nevertheless, the mechanism of rupture is more important here, in other words, it is predominantly compared to the classic case.

Note that the same observations are systematically made in all two severe biaxial configurations, with very often the total failure of the samples in two parts, especially for the most complex configuration of them, the S-Bi-60°.

**Figure 8:**
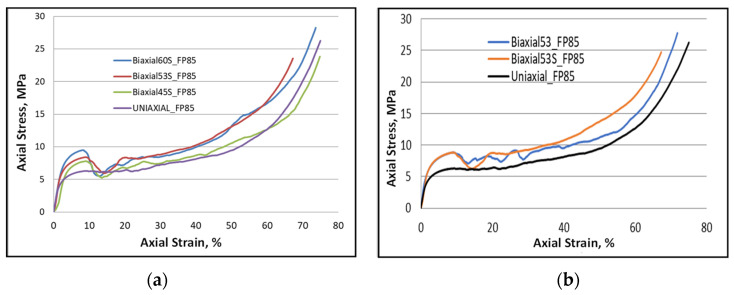
Plots showing the stress evolution versus strain for FP-85 and the impact of loading conditions on their behavior: (**a**) for S-profile, and (**b**) S-Bi-53° and Bi-53° behaviors for comparison.

To show the response of the foam under the effect of the new loading condition of compression-reverse torsion, Figure 8a illustrates the corresponding stress–strain curve. Some observations can be drawn based on this figure. Indeed, the elastic relation between stress and strain always remains as before. However, the nonlinearity which follows becomes short due to the generation of the progressive collapse of the foam cells. The compressive stress evolves continuously until reaching the maximum stress following a decrease in its value. As mentioned earlier, this is due to the collapse of a group of cells according to a given plane where the maximum stress can be found. Note that the yield and peak stresses were recorded at a point where the reverse torsional component did not reach. Figure 8b shows a comparison of foam response using the classic Bi-53° and the new S-Bi-53° loading paths.

Under the new biaxial loading configuration with severe torsion (i.e., S-Bi-53° and S-Bi-60°), the greatest strength and stress plateau for this foam was recorded as follows:

(i) The maximum shear stress generated by the ACTP rig corresponds to the maximum torsion rate given by these two loading configurations.
(ii) Additional strain hardening can be developed due to the change in twist direction, i.e., from forward to reverse. Indeed, Figure 8b shows the effect of the shear component (forward and reverse) after a value of strain of 20% where the hardening continues increasing up to the end of the load. This could be due to the dislocation multiplication within the microstructure of the employed metal generated by such a load. Such an interpretation could be based on the additional hardening induced by the biaxial cyclic loading, in particular when one of the cyclic loadings applied is a shear component [33,34]. This requires further investigation.

### 3.5. Energy Disspatation

Energy per unit volume is classically determined via the area under the stress–strain curve:

(1) W=∫σdε

where *W* is Energy per unit volume, σ is axial stress, and ε is axial strain.

**Figure 9:**
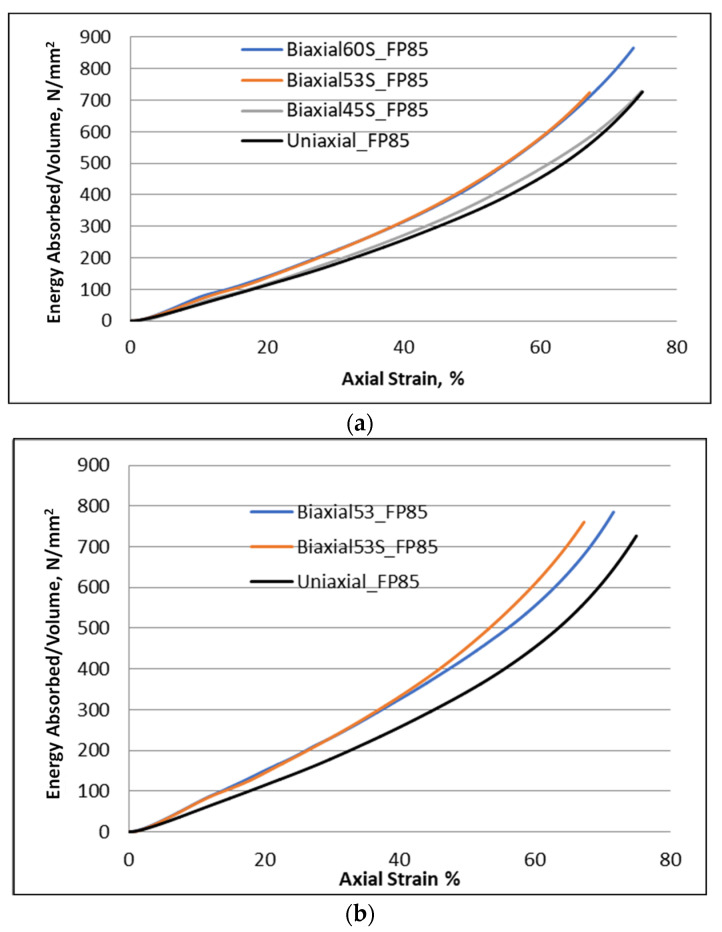

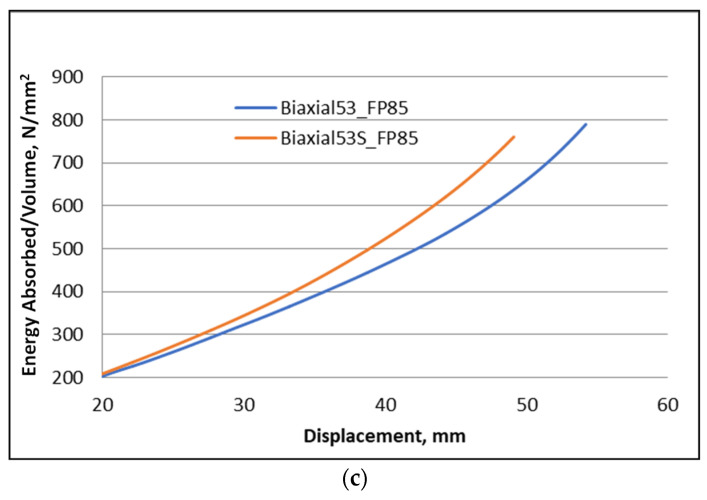
Evolution of absorbed energy per unit volume during foam collapse: (**a**) at several loading conditions, (**b**) a comparison among uniaxial, Bi-53°, and S-Bi-53° loading paths, and (**c**) a comparison between classical and new biaxial loading conditions.

For FP85 foam, Figure 9a,b revealed that the absorbed energy as a function of strain evolves in a nonlinear manner for a uniaxial loading condition. The same trend of nonlinear behavior was found for the other biaxial loads used. It was noticed that the more complex loading conditions of Bi-53° and Bi-60° led to the highest dissipated energy. Another comparison presented in Figure 9c shows that the foam loaded by the new loading condition absorbs more than the classical biaxial loading path having the same 53° inclination angle. Once again, this seems to be due to the additional hardening induced by the reverse torsional component.

One can thus conclude that the increase in the complexity of the loading generates a mode of deformation with more complex collapse (competition between the plastic deformation and the damage mechanisms). The latter leads to a greater amount absorbed energy.

**Figure 10:**
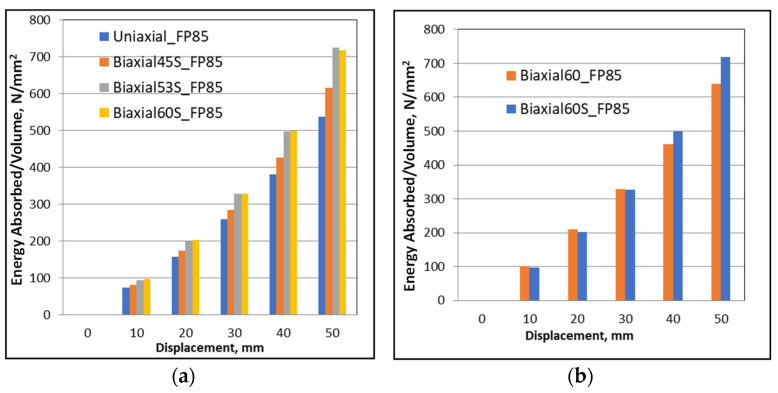
Evolution of dissipated energy for different ranges of selected displacements (**a**) along different new biaxial loading conditions, (**b**) classical rig versus the S-profiled rig.

Several displacement stages were selected presenting the evolution of the energy dissipated during the crushing process for a given load and also comparing the effect of all the biaxial loading conditions and their impact on the capacity of the foam to dissipate energy (Figure 10a). A comparison was performed targeting the effect of biaxial loading conditions and their impact on the foam’s ability to dissipate energy (Figure 10a). As expected, this capacity increases with increasing loading complexity.

The second comparison between the new biaxial (i.e., having reverse torsion) of the most complex configuration (Bi-60°) and its classic counterpart was demonstrated in Figure 10b. The new biaxial loading dissipates more than the classical one, especially for displacement greater than 30 mm.

## 4. Conclusions

The behaviors related to the yield strength, stress plateau, and dissipated energy were targeted. Local strain states were simultaneously generated in the cell struts due to the external crushing load. It depends on the applied load condition, whether uniaxial or multiaxial. In general, strain states consist of compression, bending, and buckling for uniaxial load, while an additional shear strain component is induced due to a torsional component in the case of biaxial load. The type of deformation state undoubtedly affects the mechanical properties and damages the mechanism of the foam. Therefore, the interaction between these significant mechanisms governed the foam response. One can deduce that the increase in the complexity of the loading generates a more complex mode of deformation, leading to a greater dissipation of energy from the foam structure. Reversing the torsional component during the crushing process also leads to a higher energy absorption capacity, as in the case of Bi-53° and Bi-60°.

This investigation also brings us another interesting lesson, the obvious influence of the complexity of the multiaxial loading at the microscopic scale, suggesting a certain competition between the two known plastic buckling mechanisms for this type of material, i.e., deformation and fracture, and above all a new distribution of the role of each mechanism in the plastic work. Assuming the coexistence of the two phenomena simultaneously, it is easy to observe that the share of each mechanism differs for the classical and altered biaxial situations, as was shown, for example, for the 45° biaxial configuration. Thus, it has been shown that the failure rate is significantly higher for the S-Bi-45° case, which can be physically explained by more overhardening in the basic material (aluminum).

## Figures and Tables

**Table 1:** Chemical composition of the aluminum alloy used (AS7G06) [32].

Elements	%Si	%Fe	%Cu	%Mn	%Mg	%Ni	%Zn	%Ti
AS7G06	6.7–7.3	<0.14	<0.04	<0.09	0.5–0.6	<0.04	<0.09	0.08–0.12

## Data Availability

Data will be available upon request through the corresponding author.
